# Drivers and Impacts of Postharvest Losses in Mango Production for Poverty Reduction Among Smallholder Farmers in Kintampo North Municipality, Ghana

**DOI:** 10.1002/pei3.70244

**Published:** 2026-11-10

**Authors:** Faizal Adams, Amos Mensah, Ayat Ullah, Mary Anne Mensah Laryea, Ebenezer Donkor

**Affiliations:** ^1^ Department of Agricultural Economics, Agribusiness and Extension, College of Agriculture and Natural Resources Kwame Nkrumah University of Science and Technology Kumasi Ghana; ^2^ Department of Economics and Development, Faculty of Tropical AgriSciences Czech University of Life Sciences Prague Prague Czech Republic

**Keywords:** agricultural value chains, farm income, generalized propensity score, mango postharvest losses, mango production, smallholder farmers

## Abstract

Mango is a key export crop that contributes to Ghana's economic growth. However, postharvest losses threaten the industry and the livelihoods of smallholder farmers and traders. Moreover, limited empirical studies assess the extent, constraints, and impact of these losses. The aim of this study was to quantify the amount of mango fruit wasted at the farm level, identify the causes of these losses, and assess their impact on farm income. Using a structured questionnaire, data was solicited from 297 mango producers from Kintampo North municipality in the transitional agroecological zone of Ghana. Disaggregating mango postharvest losses into quantitative and qualitative losses, the data show that approximately 137 kg (2.74 of 50 kg boxes) and 85 kg (1.7 of 50 kg boxes) are wasted per hectare respectively. In terms of economic value, the overall quantity of mango lost at the farm level translates into excess of Gh¢334 (US$32). Results from the Seemingly Unrelated Regression (SUR) model indicate that qualitative mango postharvest losses are significantly influenced by factors such as age, landownership, farm size, and credit access, whereas quantitative losses are associated with gender, family size, farm size, credit access, and type of laborer. Generalized propensity score matching (GPSM) analysis reveals that higher mango postharvest losses significantly reduce farm profitability, with increasing losses leading to declining profit margins. These findings highlight the need for targeted interventions, including improved credit access, farmer training, and postharvest management technologies, to reduce losses and enhance farmers' income. The study also contributes to addressing postharvest losses in ways that align with key Sustainable Development Goals (SDGs), including SDG 1 (no poverty), SDG 2 (zero hunger), SDG 12 (responsible consumption and production), and SDG 13 (climate action).

## 1 Introduction

Mango is a significant horticultural crop and a key nontraditional export commodity in Ghana (Adams et al. 2019). The fruit plays a crucial role in food and nutrition security while supporting the livelihoods of millions of Ghanaians, particularly smallholder farmers. Beyond its socioeconomic contributions, mango cultivation fosters sustainable agroforestry systems by promoting soil conservation and carbon sequestration, thus enhancing ecosystem and climate change resilience. Although the commercial production of mango in Ghana began only in the early 2000s, the sector has grown rapidly. By 2023, the total cultivated area had reached approximately 4856.23 ha, with annual expansions of 404.69 to 809.37 ha. This growth has resulted in an annual production volume of 983,000 metric tons (Adams et al. 2019; CERATH Development Organization 2023). Mango exports generated foreign exchange earnings of over US$64.6 million in 2021 and US$50.3 million in 2020, making the crop a vital contributor to Ghana's export revenue (Ghana Export Promotion Authority (GEPA) 2022).

Despite its economic potential, the mango industry faces significant challenges, particularly postharvest losses, which lead to substantial economic losses. Key factors contributing to these losses include the oversupply of fresh fruits during the peak harvest season, poor handling practices at the farm and during transportation, limited market outlets, and inadequate industrial capacity for value addition (Ali et al. 2021; Yahia et al. 2023). Additional issues such as fruit flies and bacteria black spot infestations, inadequate access to electricity on farms, and unreliable transportation systems exacerbate the situation (Ridolfi et al. 2018; IFPRI 2018). Current estimates suggest that postharvest losses account for 30% to 50% of harvested mangoes annually, translating to economic losses of nearly US$300 million per year (Ridolfi et al. 2018; Danish‐Embassy 2023; IFPRI 2018). A regional analysis by Gaveh (2016) found that the Brong Ahafo Region experiences the highest losses (40%), followed by the Eastern Region (36%) and the Greater Accra Region (35%) in Ghana. Such levels of wastage are alarming, especially considering the potential of these losses to provide food security without requiring additional land or production costs (Adams et al. 2023).

Considering the significant impact of mango postharvest losses on the survival of mango‐dependent households in Ghana and across Africa, several studies (Akurugu et al. 2016; Tewodros et al. 2019; Adams et al. 2019; Mujuka et al. 2020; Bambalele et al. 2021; Silué et al. 2022; Fatima et al. 2023; Fauziana et al. 2023; Chikez 2023; Githumbi et al. 2024) have explored the drivers, challenges, and management strategies to mitigate the losses. Minimizing fruit exposure to direct sunlight and soil contact during harvest and packing was highly recommended (Abera et al. 2020; Fauziana et al. 2023). While chemical fungicides can prevent postharvest diseases and pest infestations, their potential negative impact on human health makes this approach less desirable (Adams, Kaechele, et al. 2024). Alternatively, natural treatments such as peppermint and lime oil coatings or hexanal applications have been shown to delay ripening and reduce spoilage (Baltazari et al. 2020; Silué et al. 2022). Value addition has also been identified as a sustainable solution to mitigate losses. Transforming mangoes into products such as juice, dried chips, and jams during periods of high supply could expand market opportunities while reducing waste (Asante and Kuwornu 2014; Oppong et al. 2019; Adams et al. 2019). For example, mango drying initiatives in the Brong Ahafo Region have been successful in extending shelf life and creating additional income streams for farmers.

Despite these strategies, Ghana and other sub‐Saharan African countries continue to experience significant mango losses, with profound economic, social, and environmental consequences (Faibil et al. 2021). Addressing this challenge requires coordinated efforts among stakeholders, including policymakers, researchers, and the private sector, to develop and scale innovative, context‐specific postharvest management practices. One of the main reasons for the persistent high levels of postharvest losses in mango production, as well as the limited effectiveness of existing management strategies, is the lack of comprehensive research on mango postharvest estimation. While previous studies have laid a foundation and provided valuable insights into the constraints and mitigation strategies for mango postharvest losses, significant gaps remain, particularly in the estimation of these losses at the farm level. Most of the existing literature predominantly focuses on quantitative losses, with minimal attention paid to qualitative losses. Quantitative losses are easier to evaluate, as they refer to the physical quantities of produce discarded due to spoilage or damage, making them unsuitable for consumption or sale. Qualitative losses, on the other hand, involve produce sold at reduced prices due to defects such as bruises, blemishes, sap burns, or other deformities caused by postharvest handling activities. Despite their significant implications for market value and farmer incomes, qualitative losses have received little empirical attention due to the challenges associated with evaluation. However, their importance cannot be overstated, as they often represent substantial economic losses for farmers. Shee et al. (2019) emphasize the need for comprehensive assessments that capture both quantitative and qualitative losses to inform effective mitigation strategies and impactful policy development.

There is also limited empirical evidence on how mango postharvest losses affect the livelihoods of farmers, particularly regarding their revenue and economic performance. Understanding the extent of these impacts is crucial for farmers and stakeholders in adopting innovative strategies to minimize waste. Against this backdrop, the main aim of this study was to investigate the drivers and impacts of mango postharvest losses on smallholder farmers in Ghana's transitional agroecological zone. Specifically, we estimate both qualitative and quantitative postharvest losses at the farm level, assess their constraints and determinants. We further examined the impact of the losses on the income of farmers.

This study makes important contributions to both academic literature and practical approaches to addressing agricultural postharvest losses. First, we provide a holistic methodology for evaluating mango postharvest losses at the farm level. To the best of our knowledge, no previous empirical study in Ghana has simultaneously estimated both quantitative and qualitative losses, making this research a significant contribution to the field. By addressing this gap, the study offers a more complete understanding of postharvest losses and their underlying causes. Second, we extend existing methodologies by incorporating the economic valuation of mango postharvest losses, based on the framework developed by Strecker et al. (2022). This approach quantifies the monetary value of postharvest losses, emphasizing their significance to stakeholders and demonstrating the urgent need for effective mitigation measures. Third, the study adopts the Generalized Propensity Score Matching (GPSM) model to assess the impact of postharvest losses on farmers' incomes. Unlike traditional methods such as Propensity Score Matching (PSM), Endogenous Switching Regression (ESR), or Inverse Probability Weighted Adjustment Regression (IPWAR), the GPSM model allows for the evaluation of treatment variables in continuous forms. This method also effectively controls for exposure and selection biases inherent in observational studies, resulting in more reliable findings. In terms of practice, the study provides actionable insights for stakeholders in Ghana's agricultural sector. By comprehensively assessing mango postharvest losses and their socioeconomic impacts, it aims to inform evidence‐based policies and promote innovative solutions that address postharvest losses, thereby supporting sustainable agricultural development. Ultimately, the study contributes to addressing postharvest losses in ways that align with key Sustainable Development Goals (SDGs), including SDG 1 (no poverty), SDG 2 (zero hunger), SDG 12 (responsible consumption and production), and SDG 13 (climate action). By tackling mango postharvest losses, the research supports global efforts to enhance food security, reduce agricultural waste, and promote sustainable agricultural practices.

The rest of this paper is structured as follows. The conceptual framework, including the theoretical underpinnings and hypotheses, is presented in Section 2. Section 3 outlines the materials and methods employed in the study. Section 4 details the results and discussion, while Section 5 concludes with policy implications and recommendations.

## 2 Conceptual Framework

The conceptual framework for this study is grounded in the theories of profit maximization and cost minimization (Debertin 2012). These theories emphasize that farmers aim to maximize their profits by minimizing costs, including losses incurred during production and postharvest stages. In the context of mango farming in Ghana, postharvest losses significantly contribute to higher production costs, reduced revenue, and lower overall profitability. Addressing the factors driving these losses is, therefore, essential for enhancing farmers' income and economic well‐being (Aidoo et al. 2014; Arah et al. 2015; Adams, Donkor, et al. 2024).

Mango postharvest losses are influenced by three key categories of factors: personal, institutional, and farm‐related (Figure 1). Personal factors include characteristics such as age, education, family size, and farming experience. Younger farmers, for instance, may be more open to adopting innovative postharvest management technologies, while farmers with larger families may benefit from increased labor availability to minimize losses (Shee et al. 2019; Onyia et al. 2019; Savaria et al. 2020; Kulwijila 2021; Nohamba et al. 2022; Adams et al. 2023).

**FIGURE 1:**
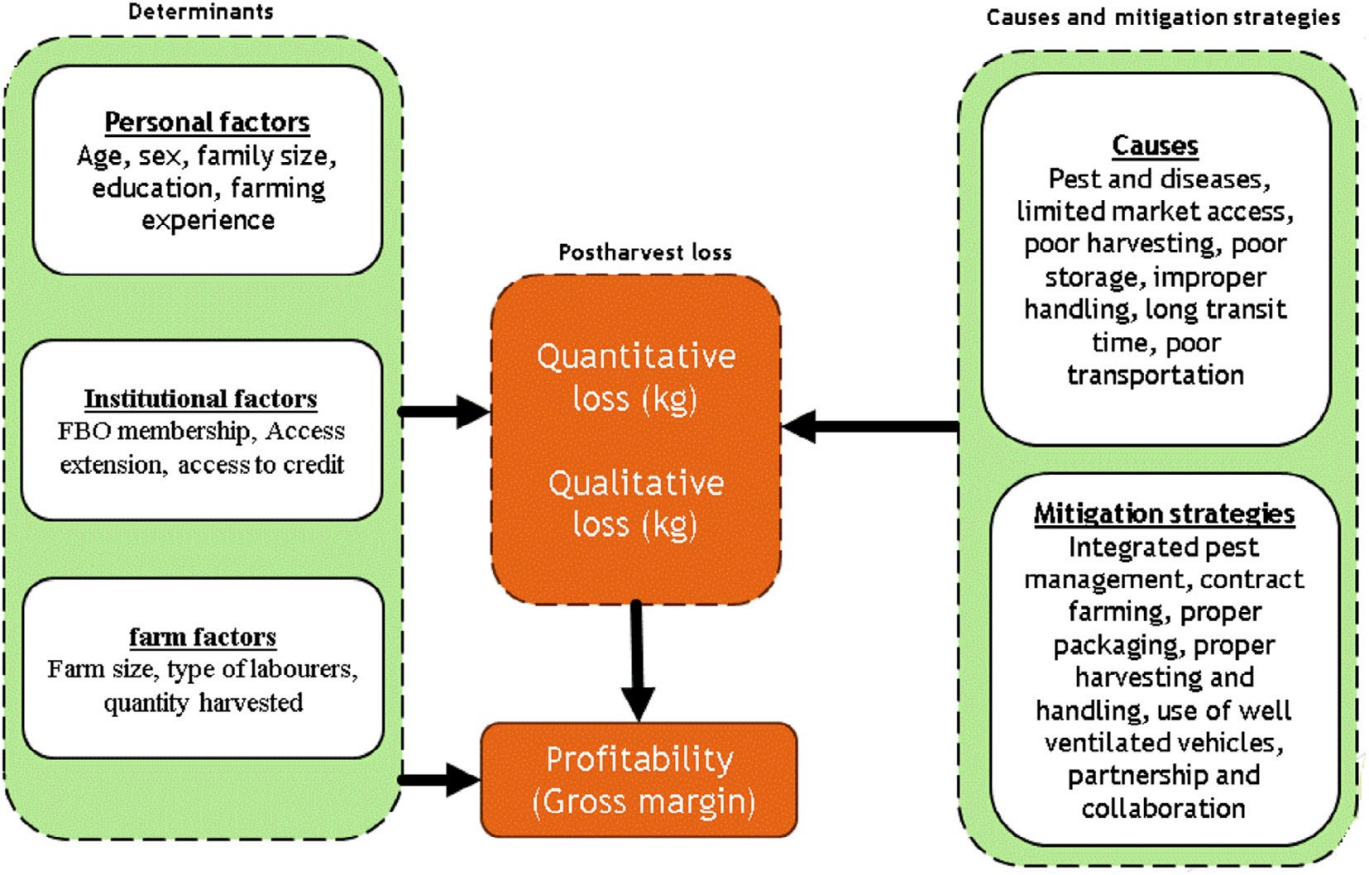
Conceptual framework of the study.

Institutional factors, such as access to credit, extension services, and membership in farmer‐based organizations (FBOs), also play a pivotal role in determining the extent of postharvest losses. Access to affordable credit enables farmers to invest in improved postharvest infrastructure, such as proper storage facilities and transportation systems. For example, mango farmers in Ghana who receive technical support from FBOs often achieve lower postharvest losses due to the collective pooling of resources and knowledge‐sharing. Similarly, extension services provide vital training on innovative postharvest handling techniques, such as the use of ventilated packaging and optimal harvesting methods, which have been shown to reduce losses significantly (Savaria et al. 2020; Wongnaa et al. 2023).

Farm‐related factors, including farm size, the quantity of mangoes harvested, land ownership, and the type of labor employed, are equally critical. Larger farms often experience logistical challenges, particularly during peak harvest periods, which can result in higher postharvest losses if not adequately managed. However, such farms are also more likely to have the financial capacity to invest in technologies, such as cold storage systems, to mitigate these losses. In contrast, smaller‐scale farmers may rely heavily on family labor, which, while cost‐effective, may not always be sufficient to handle large harvests efficiently. Additionally, the use of seasonal labor may introduce inconsistencies in postharvest practices, further exacerbating losses (Nohamba et al. 2022; Erekalo and Kelem 2022; Yimenu et al. 2024; Githumbi et al. 2024).

Understanding the interplay between these factors is essential for developing targeted interventions to reduce mango postharvest losses and improve farm profitability. The findings of this study align with the principles of profit maximization and cost minimization by identifying the critical drivers of postharvest losses and their direct impact on profitability. For policymakers, this evidence underscores the need to implement strategies such as expanding access to affordable credit facilities, strengthening extension services, and promoting FBOs to enhance the capacity of mango farmers.

These interventions can significantly reduce economic losses and contribute to the long‐term sustainability of mango farming systems in Ghana. By mitigating postharvest losses, farmers can optimize resource use, achieve better livelihoods, and strengthen their resilience to market fluctuations and environmental challenges. Such measures will not only ensure the profitability of mango farming but also support the broader goals of sustainable agricultural development in Ghana.

## 3 Materials and Methods

### 3.1 Study Area

The study was conducted in the Kintampo North Municipality (Figure 2), located in Ghana's transitional agroecological zone. Situated in the heart of the country, the municipality spans an area of approximately 4548 km^2^. Its central location and proximity to major transportation routes, including those linking Ghana to Burkina Faso, make it a strategic hub for agricultural production and trade. Kintampo's tropical savannah climate, characterized by a bimodal rainfall pattern from March to October and a dry season from November to February, supports diverse agricultural activities. Average annual temperatures range between 24°C and 35°C, coupled with moderate rainfall and fertile soils, creating ideal conditions for cultivating mangoes and other crops.

**FIGURE 2:**
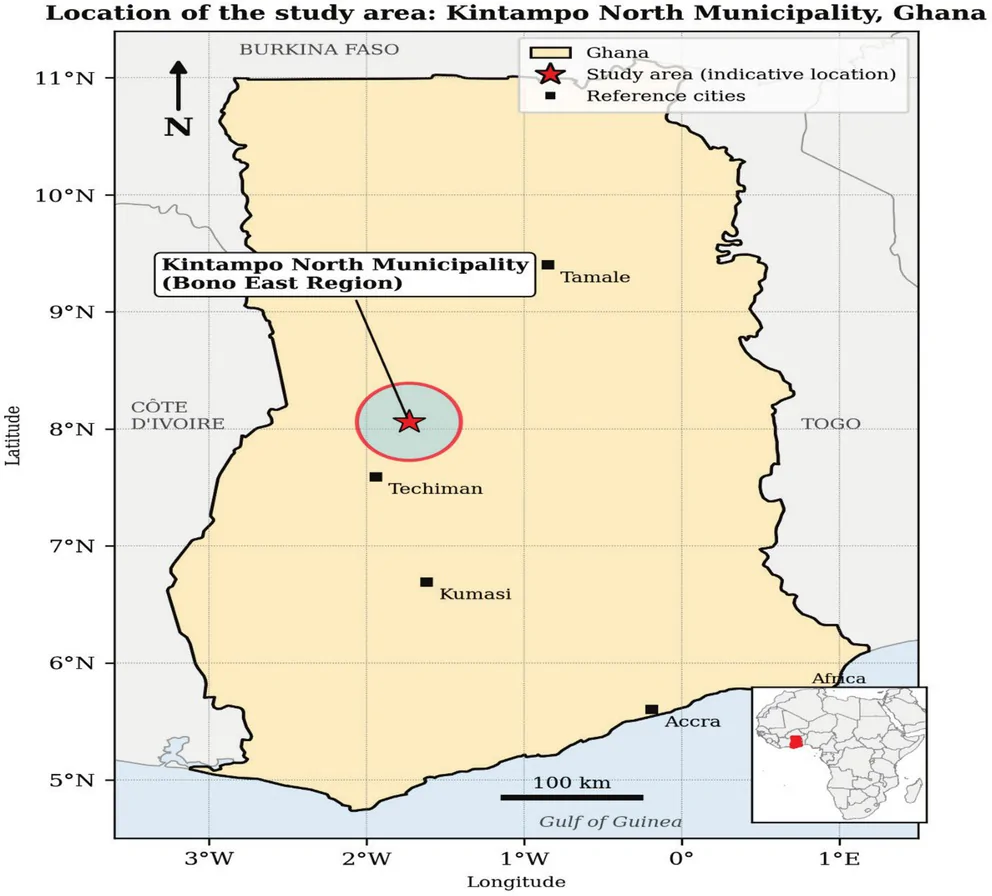
Map of Kintampo North municipality.

The economy of Kintampo North Municipality is predominantly agrarian, with about 80% of the population engaged in smallholder farming. Tree crops, particularly mango, cashew, citrus, and avocado, play a vital role in the agricultural landscape of Kintampo North. Among these, mango and cashew stand out as the most extensively cultivated. Nearly 30% of the mangoes produced in the Bono East Region originate from this municipality, underscoring its importance as a key mango production zone. Mango farming provides livelihoods for nearly 40% of the local population, with women playing a significant role in the mango value chain as farmers, traders, and retailers. The municipality also serves as a central distribution hub, facilitating the movement of mangoes from producers to both domestic and international markets, thereby contributing to the broader agricultural economy of the region.

This strategic combination of favorable climatic conditions, fertile soils, and access to transportation networks highlights Kintampo North's pivotal role in Ghana's agricultural sector, particularly in mango cultivation and trade. The municipality's unique positioning within the value chain not only bolsters local livelihoods but also strengthens its contribution to Ghana's agricultural exports, aligning with national efforts to promote sustainable agricultural development.

### 3.2 Research Design and Data Collection Process

The study employed a quantitative approach and descriptive research design, which provided a suitable framework for capturing measurable factors influencing postharvest losses and conducting a comprehensive analysis of the key variables involved (Stathers et al. 2020). This methodological approach allowed the assignment of numeric values to farmers' opinions and characteristics, which were elicited through a structured questionnaire. The questionnaire captured data on four key components: farmers' demographic and farming characteristics, details of mango postharvest losses, constraints faced by farmers, and the mitigation strategies they employed. To ensure reliability and validity, the questionnaire was pre‐tested in one community within the study area. Ten mango farmers were randomly selected for the pre‐test, and the findings informed necessary refinements to enhance clarity and accuracy.

The main data collection spanned 1 month, from April to May 2024, focusing on farmers' recollection of activities from the immediate past year. The ability of respondents to recall accurately was critical to obtaining reliable data. A sample size of 297 respondents was determined using the Yamane (1967) formula, with a 5% margin of error, from a population of 1156 mango farmers (Umar and Wachiko 2021). A multistage sampling technique was employed for selecting the respondents. At the first stage, the Kintampo North Municipality was purposively selected due to its significance in mango production and marketing in Ghana. In the second stage, five communities with high levels of mango production (Adomana‐Kunsu, Punpuano, Badukrom, Tade Fufu‐Kunsu, and Down‐Below) were purposively chosen. The number of households sampled from each community was proportionate to the population of mango farmers in each area (see Appendix Table A1). In the third stage, a simple random sampling technique was used to select mango farmers from a sampling frame obtained from the Municipality's Department of Agriculture, employing the Excel ‘rand’ command.

The data collection exercise was conducted by three teams, each comprising four final‐year undergraduate students and one supervisor. The students underwent training in questionnaire administration and actively participated in the pre‐testing phase, equipping them with the skills required for effective data collection. Each enumerator used an Android tablet equipped with the Kobo Collect application for data collection during the main survey. This ensured efficiency and accuracy in recording responses, minimizing errors associated with manual data entry.

### 3.3 Analytical Procedure

#### 3.3.1 Quantitative and Qualitative Estimation of Mango Postharvest Losses

To assess mango postharvest losses at the farm level, we adopted a subjective approach based on farmers' self‐reports, following a method similar to that used by Adams et al. (2023). Farmers were asked to estimate the quantity of mangoes lost during various postharvest activities, including harvesting, sorting and packaging, and transportation. Mangoes are typically packed in 50 kg boxes for sale, and the farmers provided estimates of the number of boxes lost during each activity. To improve the accuracy of these estimates and reduce inconsistencies in reporting, data collection was conducted immediately after the production season ended. This timing helped ensure the reliability of the reported figures.

We assessed both quantitative and qualitative losses for a more comprehensive understanding of mango postharvest losses. Quantitative loss was measured by counting the number of 50 kg boxes lost during the three postharvest activities. To estimate the monetary value of these losses, the number of lost boxes was multiplied by the market price of undamaged mangoes. The results were then standardized to a per‐hectare basis, providing a clear metric for understanding losses at the farm level. The formula for calculating quantitative loss is as follows:

(1) $$Q_{t}=N_{\mathrm{lt}}\times P_{m}$$

where $Q_{t}$ represents the quantitative loss, $N_{\mathrm{lt}}$ is the number of 50 kg boxes lost, and $P_{m}$ is the market price of undamaged mangoes.

In addition to quantitative losses, we also estimated qualitative losses, which refer to mangoes that sustained defects such as bruises, blemishes, sap burns, or other deformities during postharvest handling. These fruits are not discarded but are instead sold at a reduced price to livestock farmers or for human consumption. The price difference between the actual selling price and the ideal market price for undamaged mangoes reflects the economic loss to farmers. To calculate the qualitative loss, we first determined the number of 50 kg boxes of mangoes with defects, then multiplied this number by the price difference between the actual and ideal prices. The formula for calculating qualitative loss is:

(2) $$Q_{l}=N_{\mathrm{ll}}\times E_{l}$$

where $Q_{l}$ represents the qualitative loss, $N_{\mathrm{ll}}$ is the number of 50 kg boxes of deformed mangoes, and $E_{l}$ is the economic loss, representing the difference between the actual price and the ideal price of the defective mangoes (Strecker et al. 2022). This estimation approach provides a more holistic view of mango postharvest losses by capturing both the quantity and the economic impact of losses due to defects and handling inefficiencies.

#### 3.3.2 Seemingly Unrelated Regression Model (SUR)

We employed the Seemingly Unrelated Regression (SUR) model to investigate the factors influencing both qualitative and quantitative postharvest losses. Since these two postharvest indicators are measured as continuous variables, their residuals may be correlated when estimated simultaneously. This interdependence can violate the assumptions of Ordinary Least Squares (OLS) regression, making it unsuitable for independent estimation of each loss type (Manu et al. 2023). To address this, the SUR model is ideal, as it accounts for the simultaneous relationship between the residuals, recognizing that the likelihood of experiencing qualitative and quantitative losses is not mutually exclusive. In our study, the SUR model consists of two equations, with the following structural form that satisfies the standard regression assumptions:

(3) $$\;\left(\begin{array}{c} Y_{1}\\ Y_{2} \end{array}\right)=\left(\begin{array}{cc} X_{1} & 0\\ 0 & X_{2} \end{array}\right)\;\left(\begin{array}{c} \beta_{1}\\ \beta_{2} \end{array}\right)\;\left(\begin{array}{c} \varepsilon_{1}\\ \varepsilon_{2} \end{array}\right)$$

where $Y_{i}$ and $\varepsilon_{i}$ are N × 1 vectors, and $X_{i}$ is an N × K matrix.

We empirically estimated the SUR regression for the two postharvest loss equations as follows:

(4) $$\begin{array}{c} Y_{1}=\alpha_{0}+\alpha_{1}{\mathrm{sex}}_{1}+\alpha_{2}{\mathrm{age}}_{2}+\alpha_{3}{\text{marital status}}_{3}\\ +\alpha_{4}{\text{household size}}_{4}+\alpha_{5}\text{household}\;{\text{land size}}_{5}\\ +\alpha_{6}{\text{education}}_{6}+\alpha_{7}{\text{farm size}}_{7}+\alpha_{8}{\text{market info}}_{8}+\alpha_{9}{\text{experience}}_{9}\\ +\alpha_{10}\mathrm{fbo}\;{\text{membership}}_{10}+\alpha_{11}{\text{extension service}}_{11}\\ +\alpha_{12}\text{access}\;{\text{to credit}}_{12}+\alpha_{13}\text{type}\;{\text{of labor}}_{13}\\ +\alpha_{14}\text{quantity}\;{\text{of harvest}}_{14}+\varepsilon_{1} \end{array}$$

(5) $$\begin{array}{c} Y_{2}=\alpha_{0}+\alpha_{1}{\mathrm{sex}}_{1}+\alpha_{2}{\mathrm{age}}_{2}+\alpha_{3}{\text{marital status}}_{3}\\ +\alpha_{4}{\text{household size}}_{4}+\alpha_{5}\text{household}\;{\text{land size}}_{5}\\ +\alpha_{6}{\text{education}}_{6}+\alpha_{7}{\text{farm size}}_{7}+\alpha_{8}{\text{market info}}_{8}\\ +\alpha_{9}{\text{experience}}_{9}+\alpha_{10}{\text{fbo membership}}_{10}\\ +\alpha_{11}{\text{extension service}}_{11}+\alpha_{12}\text{access}\;{\text{to credit}}_{12}\\ +\alpha_{13}\text{type}\;{\text{of labor}}_{13}+\alpha_{14}\text{quantity}\;{\text{of harvest}}_{14}+\varepsilon_{2} \end{array}$$

In these equations, the dependent variables $Y_{1}$ and $Y_{2}$ represent the quantitative and qualitative postharvest losses, respectively. The independent variables include sociodemographic factors such as sex, age, marital status, household size, and educational level, as well as farm‐specific variables like farm size, household land size, access to credit, and type of labor used. Additionally, factors such as market information, FBO (Farmer‐Based Organization) membership, extension service access, and experience in mango farming are also included to understand their influence on postharvest losses. This approach enables us to capture the interrelated effects of these factors on both types of losses simultaneously, offering a more accurate and robust analysis of the determinants of mango postharvest losses.

#### 3.3.3 Generalized Propensity Score Matching (GPSM)

In this study, we employed the generalized propensity score matching (GPSM) to estimate the distributional impact of postharvest losses on farmers' income. The GPSM is an extended version of the standard propensity score matching (PSM) proposed by Rosenbaum and Rubin (1983). Hirano and Imbens (2004) proposed the GPSM technique for the purpose of assessing continuous treatment effects, in contrast to conventional treatment effect models that are better suited for categorical or binary treatment variables. Similar to the case of the PSM model, the GPSM has been employed in quasi‐experiments to mitigate bias arising from non‐random treatment assignments (Hirano and Imbens 2004; Bia and Mattei 2007). This technique is employed to estimate the effect of mango postharvest losses (treatment variable), measured in kilograms as a continuous variable. The estimation procedure follows a three‐step approach (Mahama et al. 2018). In step one, the generalized propensity score (GPS) is estimated as a function of the treatment and covariates. This is denoted as:

(6) $$S=s\left(T,X\right)$$

where *S* is the GPS, *T* is the treatment (Postharvest losses levels), and *X* is a vector of covariates, *s*(*T*, *X*) is the conditional density of the treatment given the covariates. The density function can be mathematically denoted as:

(7) $$s\left(T,X\right)=f_{\frac{T}{X}}\left(\frac{t}{x}\right)$$

The conditional density is divided into strata with the same values and within each stratum, it is assumed that the probability of *Y = y* is independent of *X*. This assumption is referred to as the balancing property in standard propensity score modeling. The balancing property and the assumption of confoundedness regard the treatment as being a random treatment (Mahama et al. 2018).

The second step involves the estimation of the conditional expectation of the outcome, in this case, gross profit. Based on the assumptions, the conditional expectation of the outcome is estimated using the treatment and the GPS to eliminate the estimation bias due to differences in covariates (Bia and Mattei 2008). The conditional expectation of gross profit is estimated as a function of postharvest loss levels and the estimated GPS, S. This is specified as:

(8) $$\beta \left(t,s\right)=E\left\{Y\left(t\right)|s\left(t,X\right)=s\right\}=E\left(Y|T=t,S=s\right)$$

From Equation (3), the dose function is specified as:

(9) $$\mu \left(t\right)=E\left[\beta \left\{t,s\left(t,X\right)\right\}\right],\mathrm{t\epsilon T}$$

where $\beta \left(t,s\right)$ is the conditional expectation of maize yield.

We estimated the dose–response function from the different levels of the treatment from the 1st, 2nd, 3rd and 4th quartiles by taking an average of the conditional expectation, $\hat{\beta }\left\{t,s\left(t,X\right)\right\}$, estimated from the initial step over the treatments', *t* levels. The estimated dose‐function $\mu \left(t\right)$ cannot be casually interpreted (Hirano and Imbens 2004; Bia et al. 2009), but when divided by the covariates, it yields dose‐function of the treatment, *t*. According to Serrano‐Domingo and Requena‐Silvente (2013), when the treatment dose‐function is compared with another level, $t^{\prime },\mu \left(t^{\prime }\right)$, it has a causal it takes a causal reference. Following the estimation of Mahama et al. (2018), the outcome based on the estimated parameters are specified as:

(10) $$\begin{array}{c} \hat{\mu \left(t\right)}=\frac{1}{N}\sum_{N=1}^{N}(\hat{b_{0}}+\hat{b_{1}t}+\hat{b_{2}}\log\left(\hat{p\text{score}}\right)\\ \;+\hat{b_{3}}t^{2}+\hat{b_{4}}{\left(\log\left(\hat{p\text{score}}\right)\right)}^{2}+\hat{b_{5}}\left(\log\left(\hat{p\text{score}}\right)t\right) \end{array}$$

The PSM technique is a model employed for causal inference, accounting for selection bias. It is utilized in scenarios where the treatment variable is binary. However, in the present study, the treatment variable (postharvest loss) is continuous, thus precluding the application of PSM. The GPSM model is an extended version of PSM that allows for treatments that are continuous if they have multiple categories. For this reason, the GPSM model was selected.

#### 3.3.4 Description of Variables Used in the SUR and GPSM Models

Table 1 summarizes the definitions and measurements of the independent variables included in the Seemingly Unrelated Regression (SUR) and Generalized Propensity Score Matching (GPSM) models. Previous research has shown that socio‐economic, demographic, and farm‐related factors significantly influence farming practices and productivity outcomes. For example, marital status plays a key role in the adoption of agricultural practices, as married farmers, who often have greater family responsibilities, are more likely to adopt improved practices to support their households (Tambo and Abdoulaye 2013).

**TABLE 1:** Summary of variables used in the SUR and GPSM models.

Variables	Description/unit of measurement	Expected effect
Marital status	Dummy: 1 = Married 0 = otherwise	−
Sex	Dummy: 1 = Male 0 = Female	+/−
Education	Continuous: Years in school	+/−
Farm size	Continuous: Number of hectares	+
Experience	Continuous: Number of years of farming mango	+
Market information	Dummy: 1 = Yes 0 = No	+
FBO membership	Dummy: 1 = Yes 0 = No	−
Household size	Continuous: Number of people in the household	−
Access to extension service	Dummy: 1 = Yes 0 = No	+/−
Age	Continuous: Years	+/−
Access to credit	Dummy: 1 = Yes 0 = No	+/−
Type of labor	Dummy: 1 = Full time 0 = Part time	−
Quantity harvested	Continuous: Number of 50 kg boxes	+
Household land size	Continuous: Number of hectares of land owned by the household	_
Off farm income	Dummy: 1 = Yes 0 = No	−
Land ownership	Categorical: 1 = Inherited 0 = Otherwise sources	−

*Source:* Field data 2024.

The gender of the household head also influences decision‐making in farming. Male‐headed households tend to have better access to resources such as credit, land, and market information, which may boost productivity. Conversely, female‐headed households often face resource constraints but are more likely to adopt labor‐saving practices to mitigate these challenges (Adams, Ullah, et al. 2024). Education further enhances a farmer's ability to process and apply new information, facilitating the adoption of improved agricultural practices. Educated farmers are more likely to implement new technologies effectively, which can enhance farm productivity (Asfaw et al. 2012).

Farm size is another critical factor, serving as an indicator of wealth and resource availability. Larger farms provide the opportunity for experimentation and the adoption of innovative practices, which positively impact the adoption of new technologies (Bavorova et al. 2024). Similarly, farming experience is crucial in decision‐making. Experienced farmers are better positioned to assess the risks and benefits of adopting new technologies (Caffaro et al. 2020). Access to market information further enables farmers to make informed decisions regarding planting, harvesting, and selling times, and the selection of inputs, which enhances farm productivity (Ullah et al. 2020).

Household size can have both positive and negative effects on farm productivity. Larger households may have more labor available but could also experience resource competition, which might reduce farm efficiency (Adams, Donkor, et al. 2024). Membership in Farmer‐Based Organizations (FBOs) provides access to shared resources, training, and market opportunities. However, the impact of such membership on farming outcomes depends on the structure and resources available within the organization (Ullah 2024). Access to extension services is another important factor, as it provides farmers with the knowledge and technical skills needed to adopt best practices. However, the quality of extension services received can influence the effectiveness of this support (Adams, Donkor, et al. 2024).

Age is a demographic factor that influences the adoption of farming practices. Older farmers may prefer traditional methods, while younger farmers are often quicker to adopt new innovations (Bavorová et al. 2023). Access to credit allows farmers to invest in inputs, technologies, and labor, thus improving productivity, although poor management of credit can lead to inefficiencies (Ullah et al. 2024). The type of labor used—whether full‐time or part‐time—also affects farm productivity. Full‐time laborers are typically more consistent and committed to improving farm operations, leading to higher productivity (Bavorová et al. 2023).

The quantity of harvest directly measures productivity, with higher yields indicating better farm management practices (Adams, Donkor, et al. 2024). Household land size, another indicator of wealth and operational scale, is expected to positively influence farm productivity, allowing for diversification and specialization (Bavorova et al. 2024). Off‐farm income can influence farming outcomes in varying ways. While it provides additional resources for investment, it may also divert labor and focus away from farming activities, potentially reducing farm productivity (Bavorová et al. 2023). Lastly, land ownership type affects the security of tenure, with farmers who have secure land rights, such as those owning or inheriting land, being more likely to make long‐term investments in their farms (Gorgan et al. 2024).

### 3.4 Ethical Approval and Consent

This study is based on the master's thesis research conducted at the Department of Agricultural Economics, Agribusiness and Extension, College of Agriculture and Natural Resources, Kwame Nkrumah University of Science and Technology (KNUST), Kumasi, Ghana. In view of this, the research proposal and instruments underwent review and approval by the Departmental Board of Studies and the College Board, ensuring adherence to the ethical guidelines and regulations of KNUST for research involving human participants. All procedures were carried out in accordance with these institutional requirements and the ethical principles of voluntary participation, confidentiality, and respect for respondents. Further, the study received ethical approval in accordance with the principles of the Declaration of Helsinki and institutional guidelines for research involving human subjects.

To ensure voluntariness and confidentiality, we solicited explicit oral consent from all respondents using a well‐designed process. Farmers were allowed to freely participate in the research without any coercion by providing oral consent prior to the start of the survey. The research team explained in detail the aim of the study, the types of data to be collected, and assurances of anonymity and confidentiality. This transparency allowed participants to make informed decisions about their involvement and ensured they understood the study's purpose and their role in it. Respondents were given sufficient time to complete the questionnaire, enabling them to provide thoughtful and accurate responses. Before data collection began, respondents were reassured that all information shared during the interviews would be kept confidential. The data were anonymized and reported in aggregate form to prevent the identification of individuals in subsequent analyses, publications, or presentations.

## 4 Results and Discussion

### 4.1 Socioeconomic Characteristics of the Respondents

Table 2 reveals that a significant majority of the respondents are male (79%), consistent with the findings of Okorley et al. (2014), who noted a male dominance in mango farming in the Dangme West District of Ghana. Similarly, a large proportion of the farmers were married (96%), with an average age of 55 years. The farmers also had relatively large family sizes, averaging six members, which is a crucial source of labor for smallholder farms. The education level of the respondents was fairly high, with an average of 11.6 years of formal education, suggesting a moderate capacity for adopting new farming techniques and innovations.

**TABLE 2:** Socioeconomic characteristics of the respondents.

(a) Categorical variable	Frequency	Percentage
Sex		
Female	62	20.90
Male	235	79.10
Marital status		
Otherwise	12	4.00
Married	285	96.00
Off‐farm income		
No	197	33.70
Yes	100	66.30
Household head		
No	84	28.28
Yes	213	71.72
Land acquisition		
Inherited land	211	71.00
Other sources	20	7.00
Own land	66	22.20
Access to Mkt Info.		
No	236	79.50
Yes	61	20.50
FBO membership		
No	55	18.50
Yes	242	81.50
Extension service		
No	243	81.80
Yes	54	18.20
Credit access		
Yes	234	78.80
No	63	21.20

(b) Continuous variable	Obs.	Mean	SD	Min	Max
Age of respondent (years)	297	54.98	7.225	41	68
Household size	297	5.717	1.279	1	10
Years in school	297	11.657	7.524	0	24
Experience (years)	297	13.987	5.279	5	30
(years)	297	7.704	3.36	2	16
Mango farm size (ha)	297	3.84	2.29	0.81	24.28

*Source:* field data 2024.

The data also indicated a mean farm size of 3.84 ha, with the majority of land (71%) being inherited. This aligns with the typical land acquisition patterns observed among smallholder farmers in Ghana, where land inheritance is the primary means of gaining access to farming land. In terms of income, more than half of the respondents (66.3%) supplemented their mango production with non‐farm income sources, which reflects the common practice among smallholder farmers of diversifying income sources to mitigate risks and ensure financial stability. However, despite this diversity of income sources, only 20% of the farmers had access to marketing information, highlighting a significant information gap in the sector. This is concerning given that over 80% of the respondents belong to at least one farmer‐based organization (FBO), which is expected to play a key role in information dissemination. This suggests that FBOs may not be functioning effectively in providing market‐related information to farmers. Furthermore, access to credit and extension services was limited, with only 21% and 18% of farmers, respectively, benefiting from these services. This limitation in access to critical support services may hinder the farmers' ability to invest in improvements and adopt best practices, further exacerbating the challenges they face in improving productivity and profitability.

### 4.2 Estimation of Mango Postharvest Losses

The results in Table 3 reveal a significant economic loss due to quantitative losses 137 kg (2.74 of 50 kg boxes, representing GH₵271.40) compared to qualitative losses 82 kg (1.7 of 50 kg, representing GH₵62.70), with transportation losses accounting for the highest share. This outcome aligns with expectations, as informal discussions with farmers highlighted transportation losses as the most significant. Farmers noted that they bear the costs associated with wastage during transportation, particularly when mangoes are being transported to domestic processing firms, which are the primary bulk purchasers in the region.

**TABLE 3:** Estimation of mango postharvest losses by number of 50 kg boxes per hectare in a season.

Postharvest loss stage	Quantitative loss			Qualitative loss			Overall economic loss
	(A)			(B)			
	No. of 50 kg boxes/ha	Unit price (Gh₵)	Economic loss/ha (Gh₵)	No. of 50 kg boxes/ha with qualitative defect	Price lost (Ideal‐Actual price) (Gh₵)	Economic loss/ha (Gh₵)	(A + B) (Gh₵)
Plucking	0.62 (0.305)	100 (0)	62.07 (30.90)	0.32 (0.18)	20.03 (1.91)	16.17 (9.26)	78.24 (40.16)
Gathering	0.74 (0.35)	100 (0)	74.21 (35.41)	0.44 (0.33)	20.03 (1.915)	21.93 (17.05)	96.14 (52.46)
Transportation	1.38 (0.64)	100 (0)	134.71 (66.42)	0.500 (0.35)	20.03 (1.92)	24.60 (17.28)	159.31 (83.7)
Total	2.74 (1.21)	100 (0)	271.40 (121.76)	1.7 (0.74)	20.03 (1.92)	62.70 (37.47)	334.1 (159.23)

*Note:* Numbers in parenthesis are the standard deviation, and numbers outside the parenthesis are the means. The economic value is presented in Ghanaian cedis (Gh₵), which were later converted to US dollars using the official exchange rate of US$1 = Gh₵16.35 at the time of the study.

*Source:* Field data 2024.

In total, nearly GH₵334.1 worth of mangoes per hectare are wasted during postharvest operations, representing almost 20% of the farm revenue per hectare (see Appendix Tables A2, A3, A4). This loss is consistent with previous studies, such as Gaveh (2016), which documented that mango postharvest waste in Ghana results in a revenue loss of 24% to 30%. These findings underscore the substantial economic impact of postharvest losses on smallholder mango farmers, particularly due to inefficiencies in transportation.

### 4.3 Constraints and Mitigation Strategies of Mango Postharvest Losses

The constraints and mitigation strategies for mango postharvest losses are depicted in Figures 3 and 4, respectively. Figure 3 reveals that diseases and pests (63.3%) are the leading constraints of mango postharvest losses, followed by limited access to markets (23.91%) and inadequate knowledge and skills (4.04%) related to harvesting. Fruit flies and black bacterial spots were identified as the primary pests and diseases responsible for significant economic losses in the mango industry in Kintampo and surrounding areas (Akotsen‐Mensah et al. 2017). These diseases and pests severely affect mango quality, reducing its marketability for export and creating disruptions in local supply chains. This not only diminishes the income of farmers but also affects other actors within the value chain.

**FIGURE 3:**
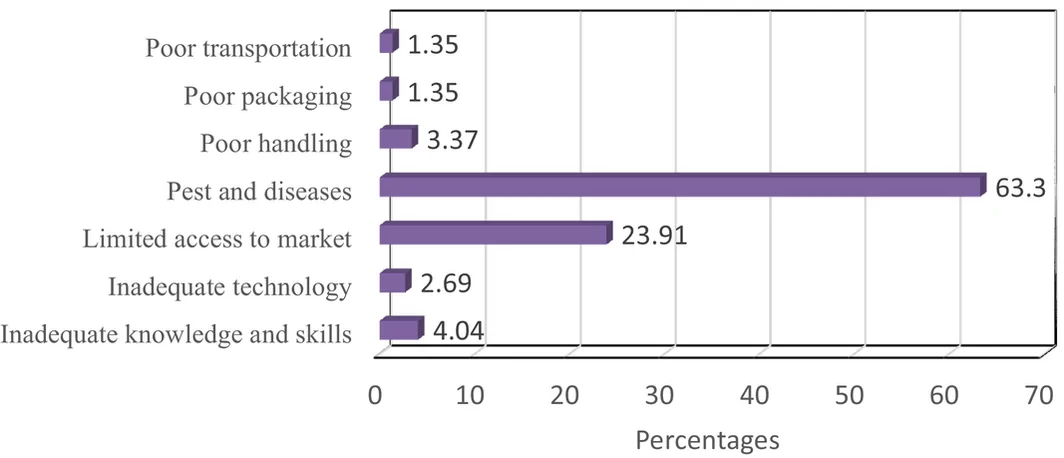
Challenges of postharvest losses. *Source:* Field data 2024.

**FIGURE 4:**
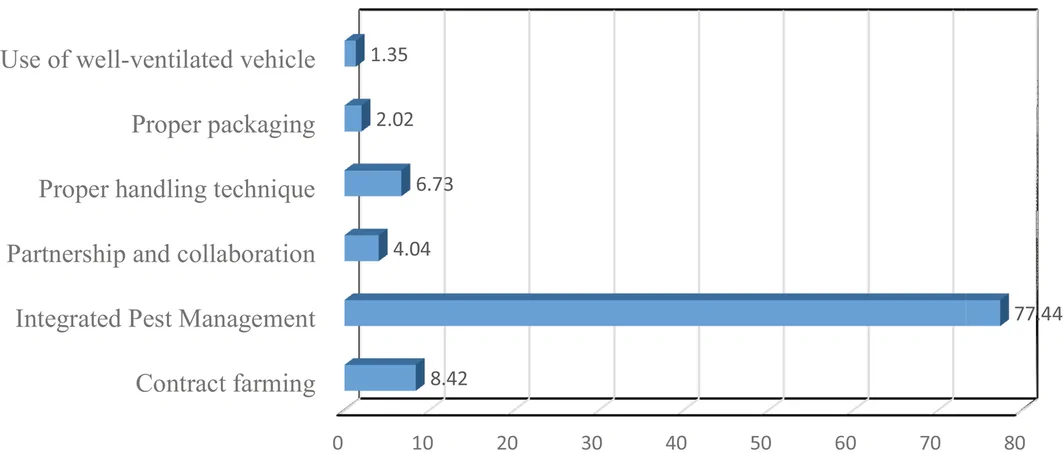
Strategies for mitigating postharvest losses. *Source:* Field data 2024.

The black bacterial spot, although it may infect mango plants before harvest, typically becomes noticeable after the fruits are harvested, complicating efforts to maintain fruit quality. Fruit flies, on the other hand, lay their larvae inside the mangoes, feeding on the pulp and rendering the fruit unfit for sale or consumption. Additionally, other pests such as anthracnose and mealy bugs were reported to accelerate postharvest losses. The finding that diseases and pests are the primary contributors to mango postharvest losses is consistent with studies by Gaveh (2016) in Ghana and Tarekegn and Kelem (2022) in Ethiopia, both of which highlight the impact of these factors on mango farming. The challenge of limited market access is closely related to poor road infrastructure, high transportation costs, and a lack of timely market information, all of which exacerbate postharvest losses. This issue is particularly pronounced in rural mango‐producing areas where farmers struggle to transport their produce to markets or processing centers due to infrastructural limitations.

To mitigate mango postharvest losses, the adoption of integrated pest management (IPM) emerged as the most commonly implemented strategy. This aligns with the finding that pests and diseases were the leading constraints of postharvest losses. Agricultural technical staff in the study area emphasized that IPM practices, such as combining good agricultural practices like pruning with the application of copper‐based substances, have been effective in controlling fruit fly populations and reducing bacterial infections. This approach offers a sustainable solution, as it decreases reliance on synthetic pesticides, which can have harmful environmental and health impacts (Chatterjee 2023).

In terms of mitigating postharvest losses, proper handling techniques ranked third, following contract farming. Many mango farmers in the study area engage in contract farming with processing companies such as Blue Sky and HPW, which helps stabilize market access and reduces the uncertainty farmers face in selling their produce. This arrangement not only ensures timely delivery of mangoes, reducing the risk of over‐ripening and decay, but also provides farmers with a guaranteed market. However, despite these positive arrangements, the use of well‐ventilated vehicles for transporting mangoes to collection points remains uncommon. This practice could significantly reduce both qualitative and quantitative losses during transportation, suggesting that the lack of attention to transportation logistics is a key factor contributing to postharvest waste.

### 4.4 Factors Influencing Mango Qualitative and Quantitative Losses

Table 4 presents the results of the seemingly unrelated regression (SUR) model used to analyze factors influencing both qualitative and quantitative mango postharvest losses. The Prusch‐Pagan Chi‐square test was employed to assess the model fit, and the significant chi‐square results at the 1% level indicate contemporaneous correlation among the residuals of the two equations. This confirms that the SUR model is the best fit for the data. Additionally, a correlation matrix among the explanatory variables revealed low correlations (< 0.5), suggesting minimal multicollinearity and supporting the inclusion of these variables in the final SUR equations (Appendix Table A6). Ten explanatory variables significantly influenced mango postharvest losses, with five related to quantitative loss (age, land ownership size, farm size, credit access, and quantity harvested) and five to qualitative loss (sex, household size, farm size, market access, and type of laborer).

**TABLE 4:** Result of SURE on determinants of quantitative and qualitative mango postharvest losses.

Variable	Qualitative loss	Quantitative loss
Sex	0.016 (0.040)	0.111** (0.049)
Age	−0.006** (0.003)	0.002 (0.004)
Marital status	−0.038 (0.087)	0.058 (0.106)
Household size	0.019 (0.015)	−0.032* (0.018)
Land ownership	−0.545*** (0.191)	0.255 (0.234)
Years of education	0.001 (0.002)	−0.003 (0.003)
Mango farm size	0.031*** (0.008)	0.040*** (0.010)
Access to market information	−0.021 (0.040)	−0.249*** (0.049)
Experience	0.001 (0.004)	0.001 (0.005)
FBO membership	−0.004 (0.041)	0.042 (0.050)
Access to extension Services	0.001 (0.043)	0.031 (0.052)
Access to credit	0.083* (0.046)	0.064 (0.056)
Type of laborer	−0.105 (0.097)	−0.578*** (0.118)
Quantity of harvest (50 kg box)	−0.001*** (0.000)	−0.000 (0.000)
_cons	8.022*** (0.291)	6.802*** (0.356)
RMSE	0.267	0.326
*R*‐squared	0.108	0.355
Chi2	35.85***	163.32***

*Note:* *n* = 297.

*Source:* Field data 2024.

* *p* < 0.1.

** *p* < 0.05.

*** *p* < 0.001.

The results show that farm size is positively correlated with both quantitative and qualitative postharvest losses. Specifically, for every additional unit increase in farm size, both quantitative and qualitative postharvest losses increase by 0.031 and 0.040 units, respectively. While larger farm sizes can generate higher incomes that may enable farmers to invest in improved technologies to reduce postharvest losses, they can also result in larger harvests that overwhelm farmers, especially during periods of market uncertainty. This leads to increased losses. This finding aligns with studies by Arends‐Kuenning et al. (2022) and Wongnaa et al. (2023), which found a positive relationship between farm size and postharvest losses.

The positive relationship between credit access and qualitative postharvest loss contradicts the findings of Akpa et al. (2023), who reported an inverse relationship between credit access and postharvest losses. This counterintuitive result may be explained by the work of Chowdhury et al. (2020), who observed that smallholder farmers often do not use credit obtained from financial institutions for improving farm operations. This suggests that while credit access could potentially facilitate investment in technologies to reduce losses, its actual utilization may not be directed toward postharvest management.

Age had a negative effect on qualitative postharvest losses, as expected. For every 1‐year increase in farmers' age, qualitative postharvest losses decreased by 0.6 units, all else being equal. This can likely be attributed to the experience and skills that older farmers have developed over time in managing postharvest losses, particularly through better harvesting and storage techniques. Similarly, household size and access to market information also had negative effects on qualitative postharvest losses. Farmers with larger families, viewed as a proxy for labor availability, can rely on additional labor to manage farm tasks such as timely harvesting and transporting mangoes to market (Essel et al. 2019). Furthermore, access to market information enables farmers to connect with a wider consumer base, reducing the risk of fruit decay and waste.

The negative relationship between land ownership and qualitative postharvest loss aligns with our expectations. Households that own land may be more motivated to invest in long‐term technologies, which can help reduce postharvest losses, including those related to fruit quality. The security of land tenure provides farmers with the confidence to make such investments, which in turn contributes to more efficient postharvest management.

The data reveals a significant association between farmers' sex and quantitative mango postharvest losses, suggesting that male farmers are more likely to incur quantitative losses compared to their female counterparts. This finding contrasts with the results of Tadesse (2020), who reported a negative effect of sex on agricultural postharvest losses, where female farmers were less likely to experience such losses. This outcome appears counterintuitive, as female farmers in developing economies often face resource constraints, limiting their ability to invest in postharvest management technologies. However, a possible explanation for this result can be drawn from Lelea et al. (2022), who noted that female farmers are more actively involved in postharvest activities such as marketing, sorting, and cleaning, which may help mitigate losses. These activities, typically performed by women, may enable better management of postharvest handling, thus reducing waste.

Furthermore, the negative effect of labor type (full time vs. otherwise) on quantitative postharvest losses suggests that farmers employing full‐time laborers are more likely to reduce losses. This makes sense, as the availability of dedicated, full‐time labor allows for the timely execution of critical tasks such as harvesting, sorting, and transporting mangoes to the market, minimizing the chances of decay and wastage. Such labor arrangements enhance the efficiency of postharvest management and contribute to a reduction in losses, as timely action is crucial to preventing fruit spoilage.

### 4.5 Effect of Mango Postharvest Losses on Farm Income

Step one of the Generalized Propensity Score Matching (GPSM) method involves estimating the GPS as a function of the treatment variable (postharvest losses) and several explanatory variables (Table 5). In the model estimation, a cube root transformation of the treatment variable was applied. The data reveal that factors such as age, marital status, land ownership, access to extension services, and use of hired labor positively influenced mango postharvest losses. Specifically, the findings suggest that as farmers age, get married, own land, have access to extension services, and hire laborers, mango postharvest losses tend to increase.

**TABLE 5:** Estimation of the generalized propensity scores.

Variables	Coefficient	SE	*p* > |*z*|
Sex	0.021	0.111	0.850
Age	0.030***	0.009	0.001
Religion	0.129	0.134	0.333
Household land size	0.393	0.606	0.516
Marital status	0.484**	0.240	0.044
Household size	−0.077*	0.044	0.079
Years of education	−0.013**	0.006	0.028
Non‐farm income source	−0.127	0.113	0.258
Land ownership	0.139**	0.057	0.016
Farm size	−0.084***	0.011	0.000
Access to marketing information	−0.315***	0.109	0.004
FBO membership	0.107	0.113	0.347
Access to extension services	0.309**	0.122	0.011
Access to credit	0.039	0.123	0.751
Source of labor	0.786***	0.268	0.003
Years of experience	−0.060	0.012	0.790
_cons	0.215	0.809	0.790
Eq2			
_cons	0.732***	0.030	0.000

*Note:* *n* = 297.

*Source:* Field data 2024.

* *p* < 0.1.

** *p* < 0.05.

*** *p* < 0.001.

The positive relationship between access to extension services and postharvest losses is counterintuitive, as extension services are generally seen as a crucial platform for smallholder farmers to learn and adopt techniques that mitigate postharvest losses (Adams et al. 2023). This contradiction could be attributed to the ineffective implementation of extension services in many African contexts, as highlighted by Adeleke et al. (2020), where extension programs may fail to reach all farmers or deliver the necessary training effectively. On the other hand, the negative relationship between family size, years of education, primary occupation, access to market information, and years of experience with postharvest losses suggests that increases in these factors contribute to a reduction in mango waste. Larger household sizes may provide additional labor, facilitating timely harvesting and better handling of mangoes (Essel et al. 2019). Similarly, higher levels of education and experience equip farmers with the knowledge and skills necessary to minimize losses. Furthermore, access to market information allows farmers to better time their harvest and sales, preventing overripe mangoes from going to waste.

The impact of postharvest loss on gross profit (Appendix Table A5) from mango production, along with its graphical representation of the dose–response, is illustrated in Table 6. The data reveal an inverse relationship between postharvest loss and farmers' gross profit. Specifically, for each unit increase in the cube root of postharvest loss, farmers experience a reduction of GH₵346.12 in their gross profit. This finding aligns with previous studies by Alidu et al. (2016) and Wongnaa et al. (2023), who also reported a negative relationship between postharvest losses and farm income. Even when adjusted for confounding factors, the negative relationship persists, reinforcing the conclusion that postharvest losses directly undermine gross revenue.

**TABLE 6:** Estimation of the impact of PHL levels (quartile levels) on gross margin using the gps model.

Gross margin	Coefficient	SE	*p*
PHL (treatment)	−346.122***	63.526	0.000
gpscores	−732.857*	416.828	0.080
PHL × gpscores	262.987	168.776	0.120
_cons	1795.782	160.379	0.000

*Note:* *n* = 297.

*Source:* Field data 2024.

* *p* < 0.05.

*** *p* < 0.001.

However, the interaction term between postharvest loss and the generalized propensity score (PHL × gpscore) is insignificant, indicating that the effect of postharvest loss on gross profit is independent of the generalized propensity scores (GPS). This suggests that the relationship between postharvest losses and gross profit remains consistent across different levels of the GPS, meaning that the impact of losses on profitability does not vary significantly based on the propensity score. This outcome implies that the factors influencing postharvest losses and the resulting reduction in profit are not moderated by other covariates captured in the GPS. Mahama et al. (2018) argued that causal inferences based on coefficient estimates are weak; instead, the significance of these estimates is more useful for identifying the presence of non‐randomness in the estimation strategy. In this study, the coefficients of both the treatment variable (postharvest losses) and the generalized propensity scores (GPScores) are significant at least at the 10% significance level, highlighting the presence of non‐randomness. This issue is effectively addressed by the generalized propensity score matching (GPSM) model, which proves to be robust in isolating the impact of postharvest losses on farm income.

The dose‐function, shown in Figure 5, graphically illustrates the impact of postharvest losses on farm income (gross margin), confirming the expected inverse relationship between these two variables. The figure emphasizes the causal link between postharvest losses and farm income, suggesting that as postharvest losses increase, farm income decreases. Figure 6 provides a more detailed analysis through the treatment dose function, which examines the marginal impact of each additional kilogram of postharvest loss on farm income (Mahama et al. 2018). The treatment dose function exhibits a decreasing rate of impact, similar to the concept of factor productivity in production economics. This pattern indicates that as postharvest losses increase from the first to the third quartile, farm income decreases from approximately GH₵1180 to GH₵1000, holding all other factors constant. However, the rate of decline sharpens at higher levels of postharvest loss. For example, an increase in postharvest loss from the third to the fourth quartile results in a significant loss of GH₵800, representing a 160% reduction in farm income. This pattern underscores the severe economic consequences of higher postharvest losses on mango farmers' profitability.

**FIGURE 5:**
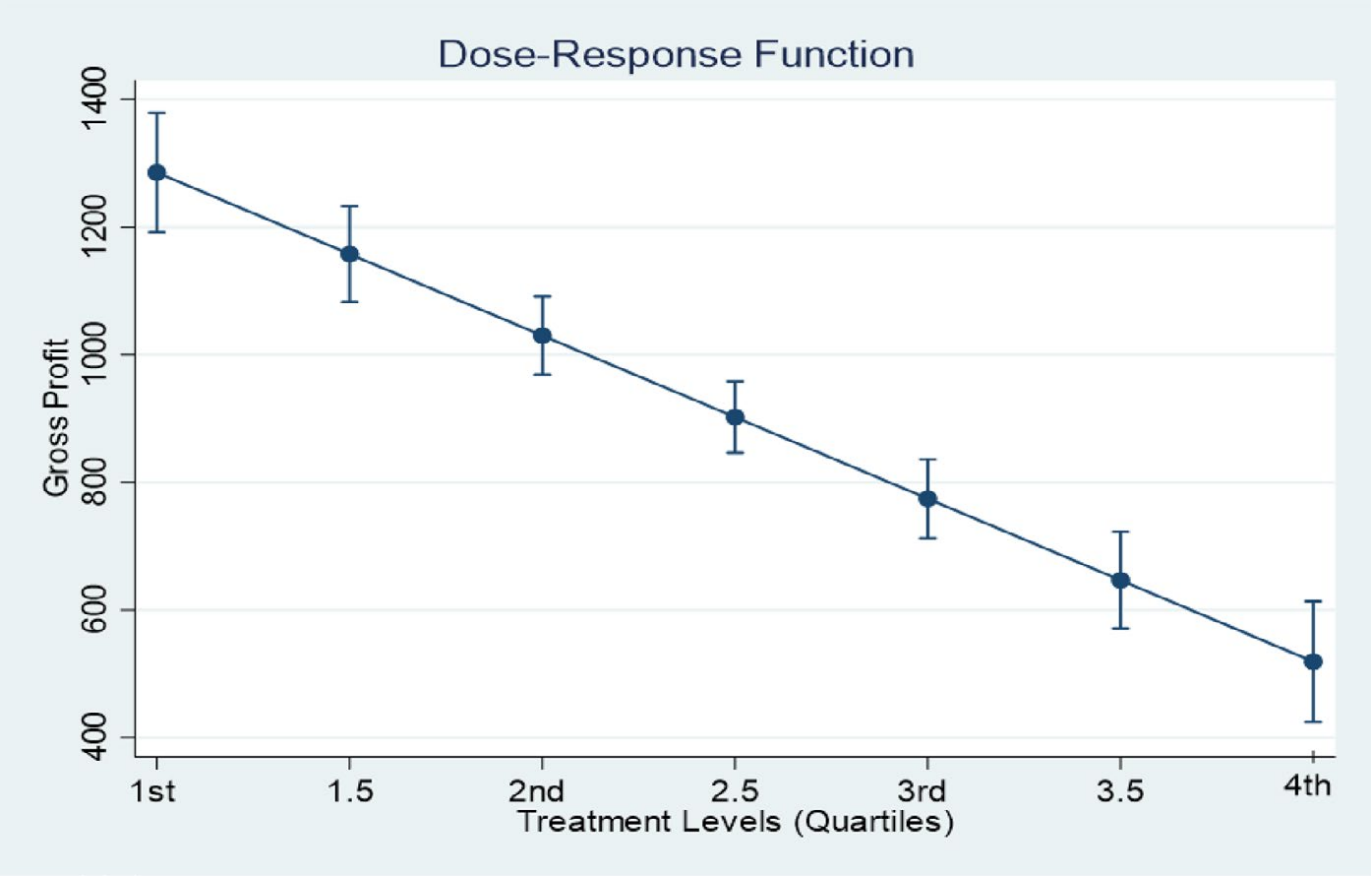
Dose–response function plots.

**FIGURE 6:**
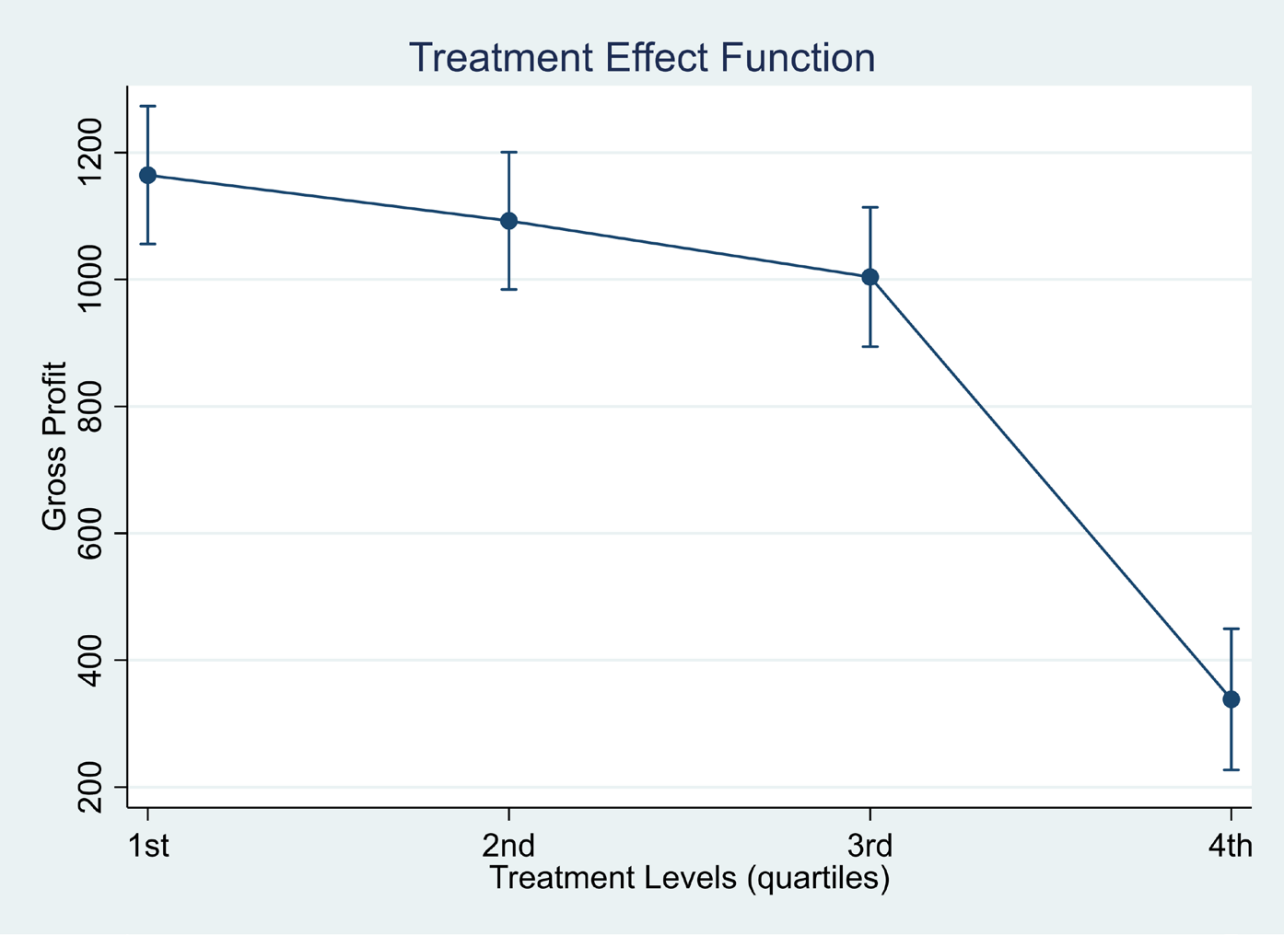
Treatment effect function plots.

## 5 Conclusion and Policy Implementation

Postharvest loss remains a significant challenge to the mango industry in Ghana. Despite its potential to alleviate poverty, improve food security, and contribute to better human health, the benefits of mango production are undermined by substantial losses along the value chain. This study, which employed the seemingly unrelated regression model to identify the factors contributing to both qualitative and quantitative mango losses, and generalized propensity score matching to analyze the impact of these losses on gross profit, contributes to the growing body of literature on sustainable agriculture in Africa. The findings confirm that mango postharvest losses are alarmingly high and are influenced by various factors, including gender, age, household size, land ownership, farm size, access to market information, access to credit, labor type (full‐time vs. part‐time), and the total quantity harvested. Additionally, the study highlights the negative impact of postharvest losses on farmers' gross margins. Notably, only 1.4% of farmers employed well‐ventilated vehicles as a strategy to manage postharvest losses, which helps explain the significant losses occurring during transportation. The study also identifies key challenges faced by the mango industry, including pests and diseases, lack of market information, inadequate storage facilities, and limited access to financing.

Based on these findings, the study recommends several policy actions. The Ministry of Food and Agriculture (MoFA), in collaboration with farmer‐based organizations and extension agents, should prioritize the regular dissemination of marketing information and training for farmers on how to access reliable market data. Such efforts can significantly reduce postharvest losses by enabling farmers to make more informed decisions. Additionally, the government should foster an enabling environment that encourages private sector involvement in postharvest loss reduction and the development of necessary infrastructure, including cold storage and transportation facilities.

To address the gender disparities in postharvest loss management, it is essential to introduce women‐focused training programs led by stakeholders such as MoFA and non‐governmental organizations (NGOs). These programs should aim to enhance women's skills in postharvest handling and management, as women are often more involved in these activities but face unique challenges that hinder their effectiveness.

## 6 Limitation of the Study

While this study provides valuable insights into quantifying postharvest losses, there are limitations that future research should address. The study's cross‐sectional design limits its ability to track changes in postharvest losses over time. Incorporating longitudinal or time‐series data would offer a more nuanced understanding of how postharvest losses evolve and enable the exploration of their long‐term impacts on the mango industry. This approach is particularly important for assessing the broader socioeconomic consequences of these losses, especially on rural livelihoods, where agriculture plays a central role in food security and economic stability. Similarly, future studies should quantify the postharvest losses in mango attributable to each contributing factor, ideally at each stage of the value chain including harvesting, sorting, packing, and transportation.

## Funding

The authors have nothing to report.

## Ethics Statement

All the procedures performed in the study involving human participants were in accordance with the ethical standards of the institutional and/or national research committee.

## Consent

Informed consent was obtained from all the individual participants involved in the study.

## Conflicts of Interest

The authors declare no conflicts of interest.

## Data Availability

The data that support the findings of this study are openly available in Zenodo at https://doi.org/10.5281/zenodo.23726750.

## Appendix A

**TABLE A1:** Communities with their household size.

No.	Communities	Household size	Number of respondents
1	Adomana‐Kunsu	218	56
2	Punpuano	167	43
3	Badukrom	261	67
4	Tade Fufu‐Kunsu	280	72
5	Down Below‐Kintampo	230	59
	Total	1156	297

**TABLE A2:** Estimation of mango quantitative PHL by number of 50 kg boxes per hectare in a season.

Items	Mean	SD
Price per 50 kg box (A)	100	0
Quantity lost		
Plucking	0.62	0.30
Gathering	0.74	0.35
Transportation	1.38	0.64
Total quantitative loss (boxes)—B	2.74	1.20
Total quantitative losses (GHS)—A × B	270.10	121.20

**TABLE A3:** Estimation of mango qualitative PHL by number of 50 kg boxes per hectare in a season.

Item	Mean	SD
Ideal price	100	0
Actual price	20.46	1.91
Price lost (A)	20.03	1.91
Quantity with qualitative defect (50 kg box)		
Plucking stage	0.33	0.18
Gathering stage	0.44	0.33
Transportation stage	0.49	0.34
Total quantity with qualitative defect (B)	1.26	0.73
Qualitative loss (GHS)—(A × B)	62.70	37.47

**TABLE A4:** Summary of mango postharvest losses by number of 50 kg boxes per hectare in a season.

Item	Mean	SD
Quantitative loss	274.18	120.16
Qualitative loss	62.70	37.47
Total PHL	336.88	145.55

**TABLE A5:** Income statement for the farmers per hectare.

Components	Amount per hectare GHs
Variable cost	
Fertilizer cost	200.11
Pesticide cost	152.89
Labor cost	322.37
Transportation cost	45.49
Total variable cost (A)	720.88
Revenue	
Mango	1770.86
Total revenue (B)	1770.86
Postharvest loss estimation (C)	
Quantitative loss	270.11
Qualitative loss	64.34
Total economic loss	334.10
Gross margin D (B − A + C)	1767.61
Less: Fixed cost	
Knapsack sprayer	140.52
Cutlass	16.01
Head pan	6.01
Pickaxe	3.85
Hoe	4.16
Annual rent	2.84
Total fixed cost (E)	173.40
Net margin F (D − E)	542.48
Total cost G (A + C + E)	1228.38
Gross margin percentage (D/B) × 100	16.37
Net margin percentage (F/B) × 100	12.41

*Source:* Field survey, 2024.

**TABLE A6:** Correlation matrix of covariates.

Variables	(1)	(2)	(3)	(4)	(5)	(6)	(7)	(8)	(9)	(10)	(11)	(12)	(13)	(14)
(1) Sex	1.000													
(2) Age	0.205	1.000												
(3) Marital status	0.063	−0.086	1.000											
(4) Household size	0.211	0.499	0.035	1.000										
(5) Household land size	−0.059	−0.057	0.017	0.018	1.000									
(6) Years of education	−0.006	−0.130	0.009	−0.087	−0.040	1.000								
(7) Mango farm size	0.058	0.266	0.018	0.279	−0.065	0.026	1.000							
(8) Access to market information	−0.046	0.070	−0.107	−0.005	−0.042	−0.018	0.044	1.000						
(9) Experience	0.049	0.356	0.038	0.387	−0.062	0.059	0.668	0.028	1.000					
(10) FBO membership	0.054	−0.033	−0.010	−0.017	0.039	0.076	−0.057	−0.187	−0.039	1.000				
(11) Access to extension services	0.092	−0.004	0.097	0.050	−0.039	−0.162	−0.081	−0.045	−0.118	−0.067	1.000			
(12) Access to credit	0.125	0.408	0.106	0.076	−0.043	−0.033	0.042	−0.019	−0.008	0.014	0.097	1.000		
(13) Type of labor	0.042	0.025	0.341	0.105	0.015	0.160	0.115	−0.044	0.021	0.103	0.088	0.006	1.000	
(14) Quantity harvested	0.027	0.253	0.038	0.222	−0.058	0.018	0.915	−0.016	0.593	−0.014	−0.119	0.153	0.152	1.000
